# A Review of *ERCC1* Gene in Bladder Cancer: Implications for Carcinogenesis and Resistance to Chemoradiotherapy

**DOI:** 10.1155/2012/812398

**Published:** 2011-10-26

**Authors:** Atsunari Kawashima, Hitoshi Takayama, Akira Tsujimura

**Affiliations:** The Department of Urology, Osaka University Graduate School of Medicine, 2-2 Yamadaoka, Suita City, Osaka 565-0871, Japan

## Abstract

The excision repair cross-complementing group 1 (ERCC1) gene performs a critical incision step in DNA repair and is reported to be correlated with carcinogenesis and resistance to drug or ionizing radiation therapy. We reviewed the correlation between ERCC1 and bladder cancer. In carcinogenesis, several reports discussed the relation between ERCC1 single nucleotide polymorphisms and carcinogenesis in bladder cancer only in case-control studies. Regarding the relation between ERCC1 and resistance to chemoradiotherapy, in vitro and clinical studies indicate that ERCC1 might be related to resistance to radiation therapy rather than cisplatin therapy. It is controversial whether ERCC1 predicts prognosis of bladder cancer treated with cisplatin-based chemotherapy. Tyrosine kinase receptors or endothelial-mesenchymal transition are reported to regulate the expression of ERCC1, and further study is needed to clarify the mechanism of ERCC1 expression and resistance to chemoradiotherapy in vitro and to discover novel therapies for advanced and metastatic bladder cancer.

## 1. Introduction

Bladder cancer is the fourth most common cancer in men in the United States [1, 2]. Bladder cancer is more prevalent in men than women, with men accounting for around 80% of cases. Up to one-half of bladder cancer cases in men and one-third in women are caused by cigarette smoking [3, 4], and another important risk factor is occupational exposure to various chemical carcinogens [5]. A common property of these exposures is the presence of carcinogens that can induce DNA damage in the bladder epithelium. Genotoxic compounds derived from the metabolism of chemical carcinogens can contribute to the accumulation of several forms of DNA damage, such as bulky adducts, single-strand breaks (SSBs) and double-strand breaks (DSBs), abasic sites, and modified bases. DNA repair mechanisms exist to prevent detrimental consequences of these types of DNA damage. Specifically, base damage, abasic sites, and SSBs are repaired through the base excision repair (BER) pathway, whereas DSBs are repaired by either nonhomologous end joining or the homologous recombination repair (HRR) pathways. Bulky adducts are generally repaired by the nucleotide excision repair (NER) pathway. An overall association was reported between genetic variation in the NER pathway and bladder cancer risk, suggesting the presence of gene-gene and gene-smoking interactions [6].

On the other hand, systemic radiotherapy and chemotherapy including cisplatin are used for locally advanced or metastatic bladder cancer, but their response rates are approximately 50%–60% [7, 8]. DNA-damaging chemotherapeutic drugs and ionizing radiation (IR) induce a variety of DNA lesions in cancer cells as well as in normal cells. The mechanisms of cisplatin and radiation resistance have been studied in various bladder cancer cell lines, and it has been shown that various DNA repair genes play important roles in resistance to various therapies [9, 10].

The excision repair cross-complementing group 1 (ERCC1) gene is located on chromosome 19q13.2-q13.3 [11]. ERCC1 performs a critical incision step in NER and is also involved in the repair of DNA interstrand crosslinks and some DSBs [12–14]. In clinical studies, the expression of ERCC1 influenced the prognosis of the patients treated with cisplatin-based chemotherapy and chemoradiotherapy (CRT) in various cancers such as lung cancer [15, 16]. Recently, several reports addressed the relation between ERCC1 expression and prognosis for cisplatin-based chemotherapy for bladder cancer [17–19], and we also reported that ERCC1 might become a good factor for predicting the efficacy of CRT for muscle-invasive bladder cancer (MIBC) [20]. In accordance with these previous reports, we review the role of ERCC1 in bladder cancer from carcinogenesis to therapeutic resistance.

## 2. ERCC1 and Carcinogenesis of Bladder Cancer

Chemical carcinogens, such as tobacco smoke and aromatic amines, can undergo metabolic activation and detoxification in the liver, and polymorphisms in the relevant genes have been shown to be associated with bladder cancer risk [21–23]. Additionally, DNA repair enzymes are required to repair the DNA damage associated with exposure to carcinogens. This suggests that common genetic variation in DNA repair genes might influence the risk of bladder cancer, and several reports examined the relation between single nucleotide polymorphisms (SNPs) in DNA repair genes and carcinogenesis. In case-control studies, several reports discussed the relation between ERCC1 SNPs and carcinogenesis in bladder cancer [6, 24–26]. Matullo et al. reported that the ERCC1-19007C variant allele (CC+CT versus TT: odds ratio (OR), 0.62; 95% confidence interval (CI), 0.41–0.95) decreased the risk of bladder cancer, which was consistent across smoking groups, although its SNP did not affect carcinogenesis in nonsmoking groups. They mentioned that the ERCC1-19007 C>T polymorphism leads to a silent Asn118Asn change, but other polymorphisms and a combination of SNPs in the *ERCC1* gene could be more important than single SNPs [24]. García-Closas et al. examined the SNPs in the NER pathway and bladder cancer risk. Compared with homozygous wild-type individuals, those carrying genotypes with variant alleles for ERCC1 IVS5+33A>C had a significant increase in risk (OR, 1.2; 95% CI, 1.0–1.5; *P*(trend) = 0.04), and the association of ERCC1 with bladder cancer risk seemed to be stronger for cigarette smokers than for never-smokers [6]. Recently, Ricceri et al. reported that a single SNP analysis showed a protective effect of the rare alleles of 3 ERCC1 SNPs: rs967591 (OR, 0.66; CI 95%, 0.46–0.95), rs735482 (OR, 0.62; CI 95%, 0.42–0.90), and rs2336219 (OR, 0.63; CI 95%, 0.43–0.93). Moreover, haplotype analysis revealed that cases of bladder cancer had a statistically significant excess of ERCC1-GAT haplotypes [26]. These reports were only case-control studies, and ERCC1 was not included in other meta-analyses [27]. From now forward, a detailed characterization of ERCC1 variation is warranted, and it is necessary to pool comparable data and identify multiple susceptibility variants that could jointly affect risk.

## 3. The Role of ERCC1 in Cisplatin and IR Resistance in Bladder Cancer In Vitro

*ERCC1* is a crucial gene in the NER pathway. Cisplatin-DNA adducts are removed via the NER pathway, and an association of different cancer cell lines with resistance to platinum compounds has been suggested [28, 29]. Welsh et al. reported that ERCC1 expression in bladder cancer cell lines was higher than that in testis tumor cell lines, and it led to less sensitivity to cisplatin-based chemotherapy in bladder cancer than that in testicular cancer [30]. We examined ERCC1 expression in four bladder cancer cell lines including two cisplatin-resistant cell lines. The cells most resistant to cisplatin had the highest ERCC1 expression, but sensitivity to cisplatin was not significantly recovered by ERCC1 knocked down by siRNA [20]. Usanova et al. reported that downregulation of ERCC1-XPF slightly but significantly increased the sensitivity to cisplatin in one bladder cancer cell line [10]. These reports indicate that ERCC1 might play a slight role in the resistance to cisplatin therapy in bladder cancer.

In regard to the association between ERCC1 and sensitivity to IR, Ahmad et al. reported that ERCC1-XPF is required for DNA DSB repair, and ERCC1-deficient cells are sensitive to IR exposure [13]. Liu et al. reported that methylation of the ERCC1 promoter correlates with radiosensitivity in glioma cell lines [31]. We reported that of four bladder cancer cell lines, the cell line with the highest ERCC1 expression was also the most resistant to IR exposure. Moreover, the sensitivity to IR exposure recovered significantly in the two cells lines in which ERCC1 was knocked down [20]. To our knowledge, there are no other reports addressing the relation between ERCC1 expression and IR resistance in bladder cancer. DSBs are the most lethal form of IR-induced DNA damage, and recent studies have observed a close correlation between the number of phospho-H2A.X foci and the number of expected DSBs after irradiation. In our study, two cell lines in which ERCC1 was knocked down recovered more slowly in terms of the number of phospho-H2A.X foci than did the control, suggesting continued accumulation or persistence of DSBs and an increase the sensitivity to IR exposure. Based on our in vitro data, ERCC1 might play greater roles in IR resistance in some bladder cancers; however, we did not show a direct correlation between ERCC1 and IR resistance in bladder cancer cells. Yacoub et al. reported that EGFR-ERK-signaling-induced IR-regulated DNA repair proteins XRCC1 and ERCC1 in prostate carcinoma cells [32]. Ko et al. reported that the level of ERK1/2 correlated with DNA repair genes, such as ERCC1 and Rad51 [33]. In clinical studies, EGFR played the key role in drug and radiation resistance [34, 35], and further study is needed to examine the difference between cisplatin and IR resistance associated with ERCC1, as well as other molecular pathways regulating ERCC1.

## 4. ERCC1 and the Efficacy of CRT for MIBC

The gold standard treatment for MIBC is radical cystectomy and urinary diversion. Concurrent CRT using cisplatin or other agents is an alternative therapy that preserves bladder function [7, 36–40]. Because the complete response (CR) rate of CRT for MIBC is 60–70%, a simple procedure is needed to select patients with MIBC who can be expected to have a good response to CRT so that they will not miss the chance to be cured by immediate cystectomy.

In terms of a predictor of the clinical response of CRT for MIBC by immunohistochemical study, Chakravarti et al. reported that in a multivariate analysis, only Her-2 expression was significantly associated with a reduced rate of CR after CRT [34]. Rödel et al. reported that the apoptotic index and Ki-67 expression, but not p53 or bcl-2 expression, were significantly related to an initial CR after CRT [41]. Matsumoto et al. suggested that only the Bax/Bcl-2 ratio was associated with the CR rate, although Bax and Bcl-2 individually were not significantly associated with the CR rate [42]. In terms of DNA repair genes, Sakano et al. studied NER, BER, and HRR SNPs in 78 patients, looking at the effect on response and prognosis following platinum-based CRT. They found the recurrence rate to be significantly lower in patients with a greater number of total variant alleles in all DNA repair genes and in NER genes, not including ERCC1 (*P* = 0.03), and the total number of variant alleles were significantly associated with improved cancer-specific survival in a univariate analysis. These findings suggested that NER genes might play an important role in the outcome of CRT for MIBC [43].

In terms of our previously mentioned in vitro data, we examined ERCC1 expression level by immunohistochemistry in all 22 patients who underwent CRT for MIBC. All patients were treated with aggressive transurethral resection of bladder tumor before CRT in Osaka University Graduate school of Medicine and its affiliated hospital. With standard fractionation (2 Gy/fraction), the median total radiation dose is 50 Gy (40–66). Moreover, concurrent platinum chemotherapy with cisplatin (*n* = 13) and nedaplatin (*n* = 9) was intravenously administered within the first and fourth week and administered doses were ranged from 100 mg to 280 mg. Six of the 8 ERCC1-positive patients showed a non-CR after CRT, whereas 12 of the 14 ERCC1-negative patients showed a CR. The efficacy of CRT as determined by ERCC1 expression level had a sensitivity of 75% and specificity of 85.7% (*P* = 0.008). Finally, we examined the correlation between ERCC1 immunoreactivity and 5-year survival in the MIBC patients undergoing CRT. Overall 5-year survival was 31.2% in the ERRC1-positive and 69.2% in the ERRC1-negative patients (*P* = 0.088). Although the sample size of our study was small, our in vitro data showed that lack of ERCC1 expression may predict the efficacy of CRT for MIBC [20].

In breast cancer, the nuclear expression of Her-2 modulated the interstrand crosslink repair of specific DNA lesions produced by chemotherapy [44]. Although we did not examine Her-2 expression in our previous study, further study is needed to examine the relation between Her-2 expression and DNA repair genes, such as ERCC1, in bladder cancer. One personalized randomized phase I/II study (RTOG-0524) was conducted using Her-2 immunohistochemistry in bladder cancer treated with radiotherapy combined with chemotherapy. The results of this clinical study warrant further investigation about the relation between them. Moreover, Fleischmann et al. recently reported that Her-2 expression in lymph node metastasis was higher than that in primary bladder tumors [45]. It will be important to assess novel CRTs targeting the Her-2/DNA repair pathway in advanced bladder cancer with metastasis.

## 5. ERCC1 and the Efficacy of Cisplatin-Based Chemotherapy

In a clinical study using immunohistochemistry, Olaussen et al. reported that patients with ERCC1-negative nonsmall cell lung cancer appeared to benefit from adjuvant cisplatin-based chemotherapy, whereas patients with ERCC1-positive tumors did not [15]. In advanced bladder cancer, Bellmunt et al. were the first to report that patients with a high mRNA level of ERCC1 had poorer prognosis for cisplatin-based chemotherapy than did patients with a low mRNA level of ERCC1 [17]. However, the authors did not make a general statement concerning the influence of ERCC1 expression on the outcome of other treatment regimens including radiotherapy. In adjuvant cisplatin-based chemotherapy for MIBC, Hoffmann et al. reported that high mRNA levels of ERCC1 and MDR1 predicted inferior progression-free survival [18]. Various regimens of cisplatin-based chemotherapy were used in these previous reports. ERCC1 expression was not different between the two platinum-based treatment arms (CMV versus M-VAC), and there was no significant relation between ERCC1 expression and progression-free survival in either therapeutic regimen (*P* = 0.21, *P* = 0.07) [18]. Matsumura et al. reported that the expression of not ERCC1 but human equilibrative nucleoside transporter 1 (hENT1) could predict prognosis in patients treated with gemcitabine and cisplatin (GC) therapy in an immunohistochemical study [46]. As the major molecular component of nucleoside transporter proteins, hENT1 has been shown to predict benefit in patients undergoing gemcitabine chemotherapy for several solid malignancies [47, 48]. Matsumura et al. reported that only hENT1 expression (*P* = 0.004) and not ERCC1 expression (*P* = 0.182) was associated with the prognosis of overall survival by multivariate analysis. Kim et al. reported that immunohistochemical expression of ERCC1 could predict only progression-free survival and not overall survival for advanced bladder cancer treated with GC and M-VAC therapies. They indicated that ERCC1 negativity was a statistically significant independent prognostic marker for progression-free survival (OR, 1.62; 95% CI, 1.03–2.54; *P* = 0.003) [19]. The role of ERCC1 as it relates to resistance to cisplatin was controversial in clinical studies of bladder cancer. Recently, we examined the prognosis of cisplatin-based neoadjuvant chemotherapy in 58 bladder cancer patients treated with M-VAC and GC therapy using the results of an immunohistochemical study of ERCC1. The expression of ERCC1 could not predict CR or the prognosis for either disease-free or overall survival (data not shown). Hsu et al. reported that Snail, one of the endothelial-mesenchymal transition (EMT) markers, regulated the expression of ERCC1, and coexpression of Snail and ERCC1 predicted the poor prognosis of cisplatin-based chemotherapy for head and neck cancer [49]. EMT is reported to play an important role in progression and resistance to radiotherapy and chemotherapy in bladder cancer and other malignancies [35, 50–53]. We previously examined Snail expression and found that it correlated with ERCC1 expression (*P* = 0.001). Moreover, coexpression of ERCC1 and Snail were also found to be the prognostic factors to predict poorer disease-free and overall survival by both univariate and multivariate analysis in all cases examined. In this study, coexpression of ERCC1 and Snail were prognostic factors only in the patients undergoing M-VAC therapy (data not shown). In these above studies, the ERK1/2-Snail/Slug pathway including tyrosine kinase receptors such as Her2 or EGFR might regulate the expression of ERCC1, and further in vitro and clinical study is needed to clarify the relation between ERCC1 and cisplatin resistance. In addition, prospective randomized trials of therapy based on expression levels of ERCC1, such as that performed in lung cancer, are needed [54].

## 6. Conclusion

We reviewed the role of ERCC1 in bladder cancer from carcinogenesis to therapeutic resistance. In bladder cancer, ERCC1 might become an important factor to predict cisplatin resistance as in other malignancies, and our in vitro results suggested that in some bladder cancer cells, ERCC1 expression correlates with resistance to IR but not with resistance to cisplatin. Moreover, lack of ERCC1 expression correlated well with the efficacy of CRT and especially with that of IR. Tyrosine kinase receptors such as EGFR and Her2 might regulate the expression of ERCC1, and EMT is closely correlated with the expression of ERCC1. Further study is needed to clarify the mechanism of the relation between ERCC1 and resistance to cisplatin and IR in vitro and to discover novel CRTs for the treatment of advanced and metastatic bladder cancers.

##  Conflict of Interests

The authors declare no conflict of interests.
